# Simple protocol for combined extraction of exocrine secretions and RNA in small arthropods

**DOI:** 10.1093/biomethods/bpae054

**Published:** 2024-07-30

**Authors:** David Fröhlich, Michaela Bodner, Günther Raspotnig, Christoph Hahn

**Affiliations:** Department of Biology, University of Graz, Graz, 8010, Austria; Department of Biology, University of Graz, Graz, 8010, Austria; Department of Biology, University of Graz, Graz, 8010, Austria; Department of Biology, University of Graz, Graz, 8010, Austria

**Keywords:** biosynthetic pathways, chemical ecology, chemosystematics, differential expression analysis, gland secretion, oribatid oil glands, phylotranscriptomics, transcriptomics

## Abstract

The integration of data from multiple sources and analytical techniques to obtain novel insights and answer challenging questions is a hallmark of modern science. In arthropods, exocrine secretions may act as pheromones, defensive substances, antibiotics, as well as surface protectants, and as such they play a crucial role in ecology and evolution. Exocrine chemical compounds are frequently characterized by gas chromatography–mass spectrometry. Technological advances of recent years now allow us to routinely characterize the total gene complement transcribed in a particular biological tissue, often in the context of experimental treatment, via RNAseq. We here introduce a novel methodological approach to successfully characterize exocrine secretions *and* full transcriptomes of one and the same individual of oribatid mites. We found that chemical extraction prior to RNA extraction had only minor effects on the total RNA integrity. De novo transcriptomes obtained from such combined extractions were of comparable quality to those assembled for samples that were subject to RNA extraction only, indicating that combined chemical/RNA extraction is perfectly suitable for phylotranscriptomic studies. However, in-depth analysis of RNA expression analysis indicates that chemical extraction prior to RNAseq may affect transcript degradation rates, similar to the effects reported in previous studies comparing RNA extraction protocols. With this pilot study, we demonstrate that profiling chemical secretions and RNA expression levels from the same individual is methodologically feasible, paving the way for future research to understand the genes and pathways underlying the syntheses of biogenic chemical compounds. Our approach should be applicable broadly to most arachnids, insects, and other arthropods.

## Introduction

Within Arthropoda, exocrine glands for communication, chemical defense, and protection have evolved multiple times. Oribatid mites, for instance, possess so-called “oil glands.” These glands represent phylogenetically old exocrine systems, with the origin of glandulate oribatids dating back to about 571 ± 37 mya (earliest Parhyposomata in [1]). Among extant Oribatida, a stunning diversity of oil gland-derived compounds is produced [2] some of which with potential biomedical applications like fungicide and bactericide effects [3, 4]. However, the biosynthetic pathways and genes involved in the production of these compounds are poorly understood and detailed investigations are only available for *Archegozetes longisetosus* Aoki, 1965 [5–7] and a few Astigmata [8, 9]. Most recently, Brückner and colleagues [7] proposed a reconstruction of the biosynthetic pathway leading to monoterpenes in *A. longisetosus* informed by a combination of mass spectrometry data of stable isotopes and genomic analyses.

Using several approaches in an integrative way has become standard in chemical ecology. Examples of recent research combining data from different sources and analytical approaches focus on integrative taxonomy [10], chemotaxonomy [11], chemosystematics and chemo-phylogeny [12–14]. Especially for chemo-phylogenetic approaches, which seek to track the evolutionary histories of secretion chemistry in particular glands, a detailed understanding of the underlying biosynthetic pathways is required.

DNA extraction of individuals after the extraction of exocrine secretions is meanwhile common [10, 14–16]. In this context, some effort has been made to elaborate DNA-extraction methods in small arthropods [17]. The extraction of chemical secretions *and* RNA from the same individual has already been reported performed [18, 19]. However, the exact procedure is not explicitly described and only the RIN value was used for quality assessment. We therefore considered a more in-depth investigation of the RNA quality and the expression levels worthwhile. In addition to whole genome sequencing, combined data on secretion chemistry and mRNAs could provide a paramount foundation for identifying relevant biosynthetic pathways for exocrine compounds and their underlying genes, respectively. After such target genes have been identified, experimental approaches may be designed to study expressed enzymes and pathways, including those responsible for the production of exocrine compounds and their temporal interlacing. Additional dissection of the animal to sequence specific tissues and scRNA-seq (single-cell RNA-seq) may also be possible.

**Figure 1.**
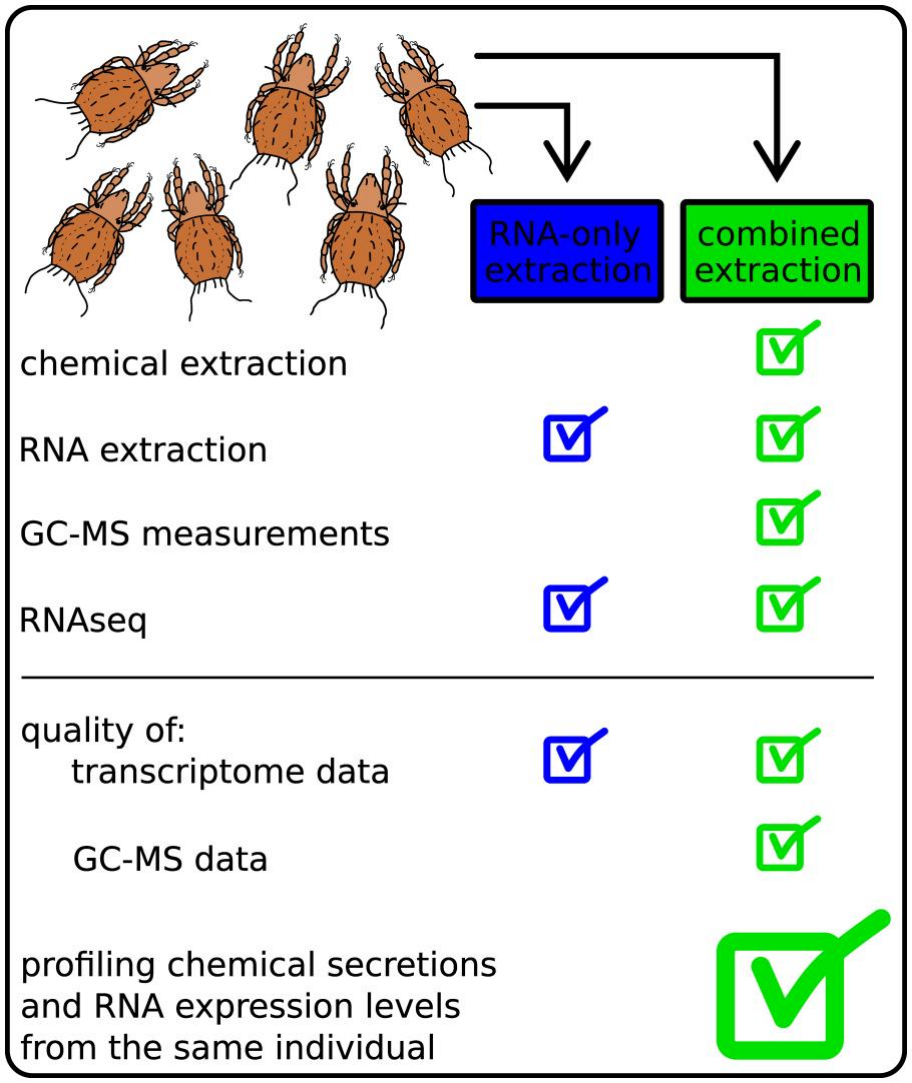
Main parts and outcomes of the present study and the design of our pilot study.

The goals of the current pilot study were (i) to test the feasibility of performing combined extractions of exocrine secretion compounds and total RNA from the same arthropod specimens and (ii) to compare RNA integrity-composition and-expression profiles of samples with and without chemical extraction prior to RNA extraction. For this study, we used individuals of *Nothrus palustris* Koch, 1839. This is a rather large (990–1200µm) oribatid mite species of the cohort Desmonomata (family Nothridae) with holarctic distribution [20]. The oil gland secretion compounds of this mite are well characterized. They were first investigated by Shimano et al. [21] and recently re-investigated [22]. Figure 1 illustrates the design of our pilot study which we consider the foundation for future experiments, specifically designed to identify the underlying genes responsible for the biosynthesis of exocrine compounds in a variety of arthropods.

## Material and methods

### Sample collection and preparation

Specimens of *N. palustris* were extracted (using a Berlese-Tullgren apparatus) from sieved litter samples collected in Heiligenkreuz am Waasen (Styria, Austria) in 2022. Material from the sampling campaign was randomly assigned into two different treatment groups: (i) RNA extraction only and (ii) combined extraction, starting with chemical extraction followed by RNA extraction. Each sample comprised three pooled individuals. Three replicates were performed per treatment. RNA extraction was performed using the Promega ReliaPrep™ RNA Miniprep System following the manufacturer’s protocol after homogenization. In brief, specimens were transferred to tubes (Beadbug™ prefilled 2-ml tubes with 1-mm triple-pure-high-impact zirconium beads) prepared with 250 µl LBA + TG Buffer for homogenization (MPI™ FastPrep^®^-24 homogenizer: 4 m/s for 20 second, repeated after 20 second). RNA dilution buffer was added and vortexed, and the samples were incubated (step 2 of the manufacturers protocol) within the Beadbug-Tube. Subsequently, the lysate was transferred into clean Eppendorf tubes. For elution, 15 µl of nuclease-free water was used and the RNA quality and quantity of samples were measured using Qubit™ RNA High Sensitivity Assay Kit and Tapestation RNA ScreenTape Analysis.

The same RNA extraction protocol was also used for group two (combined extraction), but in addition, a chemical extraction of the defensive gland secretions was performed prior to RNA extraction. Three individuals of *N. palustris* were extracted in 30 µl methylene chloride (DCM) in vials for 15 min. Vials were kept on crushed ice before and during secretion extraction. Secretion-loaded solvent was transferred into a new vial and analyzed by gas chromatography–mass spectrometry (GC–MS). Once the remnants of the solvent in the vials evaporated, the specimens were transferred into tubes for homogenization and RNA extraction as described above. The full protocol of the combined extraction can also be found at https://doi.org/10.17504/protocols.io.n92ld8mb7v5b/v1.

### RNA data processing

Stranded RNAseq library preparation (poly A enrichment) was done by Novogene UK and sequenced on an Illumina HiSeq 6000 (150 bp, paired-end). For quality control, FASTQC [23] was used and obtained data were trimmed using fastp 0.12.4 [24, 25] using standard parameters. De novo transcriptome assembly was performed for each sample individually using Trinity 2.13.2 [26] (--min_kmer_cov 2). Transcriptome completeness was tested using BUSCO 5.2.2 [27] (lineage dataset: arachnida_odb10). All these steps were performed on the high-performance computing (HPC) infrastructure of the University of Graz (GSC1) and the Vienna Scientific cluster (VSC4). For quantitative analysis, we initially employed Seq2Fun 2.0.5 [28] using their arthropods_v2.0 database, within WSL2 Ubuntu 20.04.5 LTS running under Windows 10 x64 (build 19044). The settings can be found in the supplementary file 1. The Seq2Fun approach makes use of a predefined set of reference transcripts and thus uses only a subset of transcripts due to the filtering during the mapping process. Subsequently, differential expression analysis of the resulting reduced transcriptome was performed using ExpressAnalyst [29] with DEseq2 [30] (adjusted *P*-value ≤ 0.05, logFC = 0). Further, we analyzed the data following a second strategy which is expected to be less restrictive, designated as the full-transcriptome-approach. We first assembled a single meta-transcriptome using reads from all samples conjointly using Trinity. Transcripts of individual samples were then quantified using salmon 1.9.0 [31] (--validateMappings --gcBias --seqBias) based on the meta-transcriptome. Differential expression analysis was done using tximport [32] and DEseq2 1.38.2 [30] in R 4.2.2 [33] running under Windows 10 x64 (build 19044). A pre-filtering was performed by using only transcripts with a row sum of the counts ≥ 10 and count-values for at least three samples. Transcripts were considered as differentially expressed in both cases if the adjusted *P*-value ≤ .05. For the differentially expressed gene (DEG)-analysis with the full dataset, three different thresholds of the logFC were used (0, 1, and 2). Count data have been normalized before calculating PCA (plotPCA {DESeq2}). For comparison, the analysis with the reduced set has only been performed with a logFC of 0.

### GC–MS

Aliquots (1.5 µL) of the DCM extracts from group 2 (combined extraction) were measured on an Agilent 5977B GC/MSD (Vienna, Austria). The gas chromatograph was equipped with two connected HP-5MS ultra inert capillary columns (15 m x 0.25 mm id., 0.25-µm film thickness; Agilent, Austria). Instrumental parameters were the same as recently described [34]. Generated data were processed in Agilent Mass Hunter software 10.0. For verification of the quality of the extracts prepared on ice (= group 2: Dori065, Dori066, Dori067), we compared the chromatograms with the individual profiles published by Raspotnig et al. [22].

## Results

Table 1 details the RNA concentrations, RNA integrity, as well as measures of RNAseq data quality for the different samples. No difference in the RNA concentration between groups could be found (mean concentration: 32.4 ng/µl for the combined extraction and 29.9 ng/µl for RNA-only).

**Table 1.** Amount of RNA measured with the Qubit™ RNA High Sensitivity Assay Kit. For the RNA concentration, mean values of three measurements were calculated. Reads of RNA-seq are also given before and after filtering.

Sample	Method	RNA [ng/µl]	RIN	Raw reads	Filtered reads	Transcriptome BUSCO-completeness
DOri065	Combined	39.3	8.4	18 492 913	18 260 664	97.31%
DOri066	Combined	22.5	8.7	18 85 6173	18 554 353	97.44%
DOri067	Combined	35.4	8.6	23 772 278	23 485 248	97.31%
DOri068	RNA only	28.9	9.3	21 455 672	21 175 758	92.81%
DOri069	RNA only	34.9	9.3	19 925 640	19 672 916	97.99%
DOri070	RNA only	25.9	9.8	25 934 617	25 569 929	97.75%

**Figure 2.**
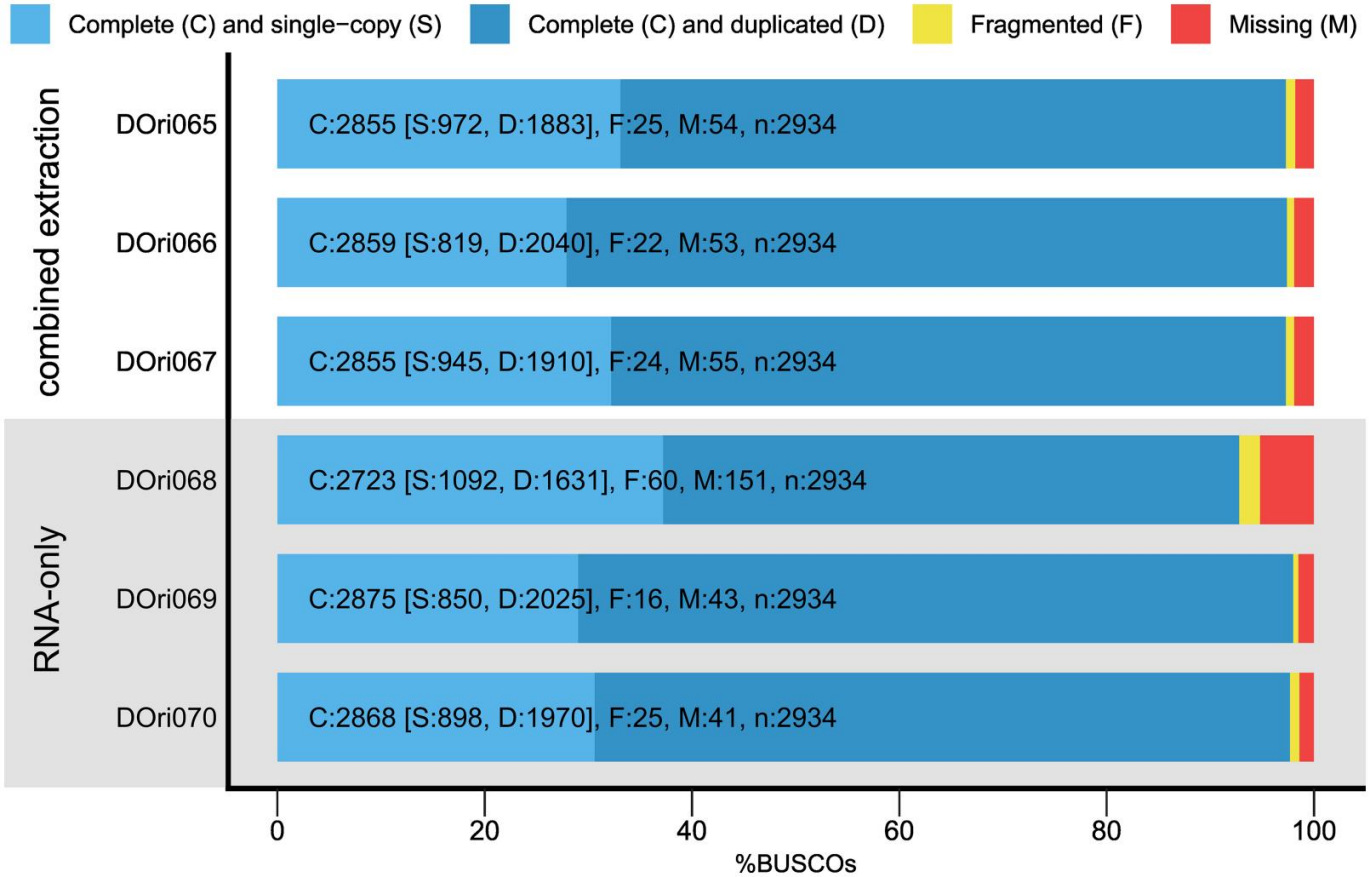
BUSCO assessment to test for completeness of de-novo assemblies of all six samples.

The quality control by Tapestation Analysis showed no difference in fragment length distribution between samples from the two groups, although the mean RIN for the RNA-only (9.5) was slightly higher compared with the combined extraction (8.6). Quality control of raw reads (FASTQC) showed no major differences before and after trimming (98.4–98.8% of the reads remained) (Table 1). The BUSCO analyses of the samples (Table 1, Fig. 2) showed an average completeness of 97.35% (combined extraction) and 95.71% (RNA-only), respectively. These results were highly consistent, with only one sample of the RNA-only group with slightly lower completeness (DOri068).

The reduced transcript dataset considered the expression of 7148 genes (total feature number), whereas the full-transcriptome-approach considered 241 454, of which 69 764 transcripts passed the initial filtering step and were included in the statistical analyses.

**Figure 3.**
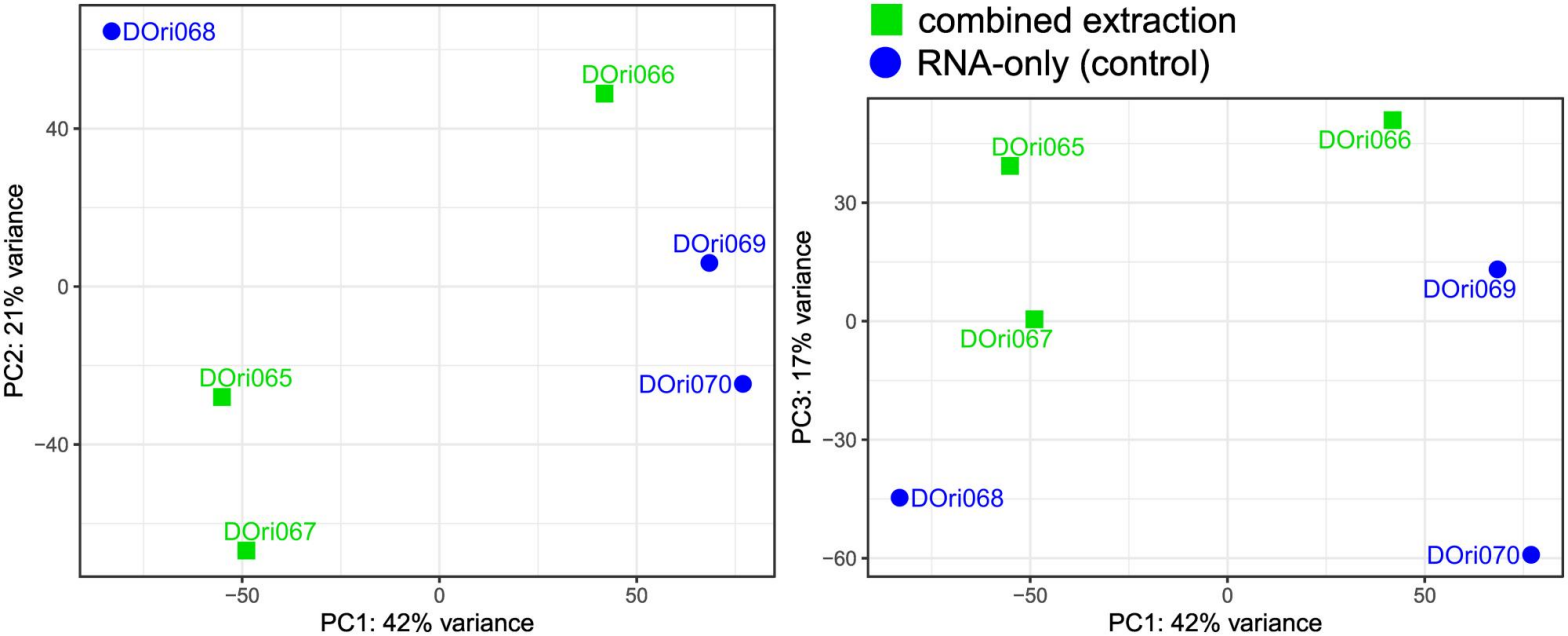
Principal components 1–3 of differential expression analysis with full-transcriptome-approach without clustering according to the extraction method used. PC 1–3 explain 80% of the total variation.

In the full-transcript-approach, no sign of by-group clustering could be found even if investigating PC 1–5 (Fig. 3, PC 4–5 not shown), while in the reduced dataset PCA indicated a clustering of the samples from the two treatments with one sample being an outlier (supplementary file 2). There, PC2 (explaining 20.1% of the variance) separates the two groups. Table 2 shows the results of the differential expression analysis of the full dataset.

**Table 2.** Results of the differential expression analysis (up- and downregulated in combined extraction) using the full dataset and three different logFC values.

	logFC = 0	logFC = 1	logFC = 2
logFC > X (upregulated)	361	147	76
logFC < X (downregulated)	1718	618	258
total number of diff. expressed transcripts	2079	765	334

The reduced dataset returned 166 DEGs (Supplementary file 3; 146 downregulated in the combined extraction group and 20 upregulated). The analysis indicated enrichment of a number of gene ontology terms in the DEGs (see supplementary file 4).

**Figure 4.**
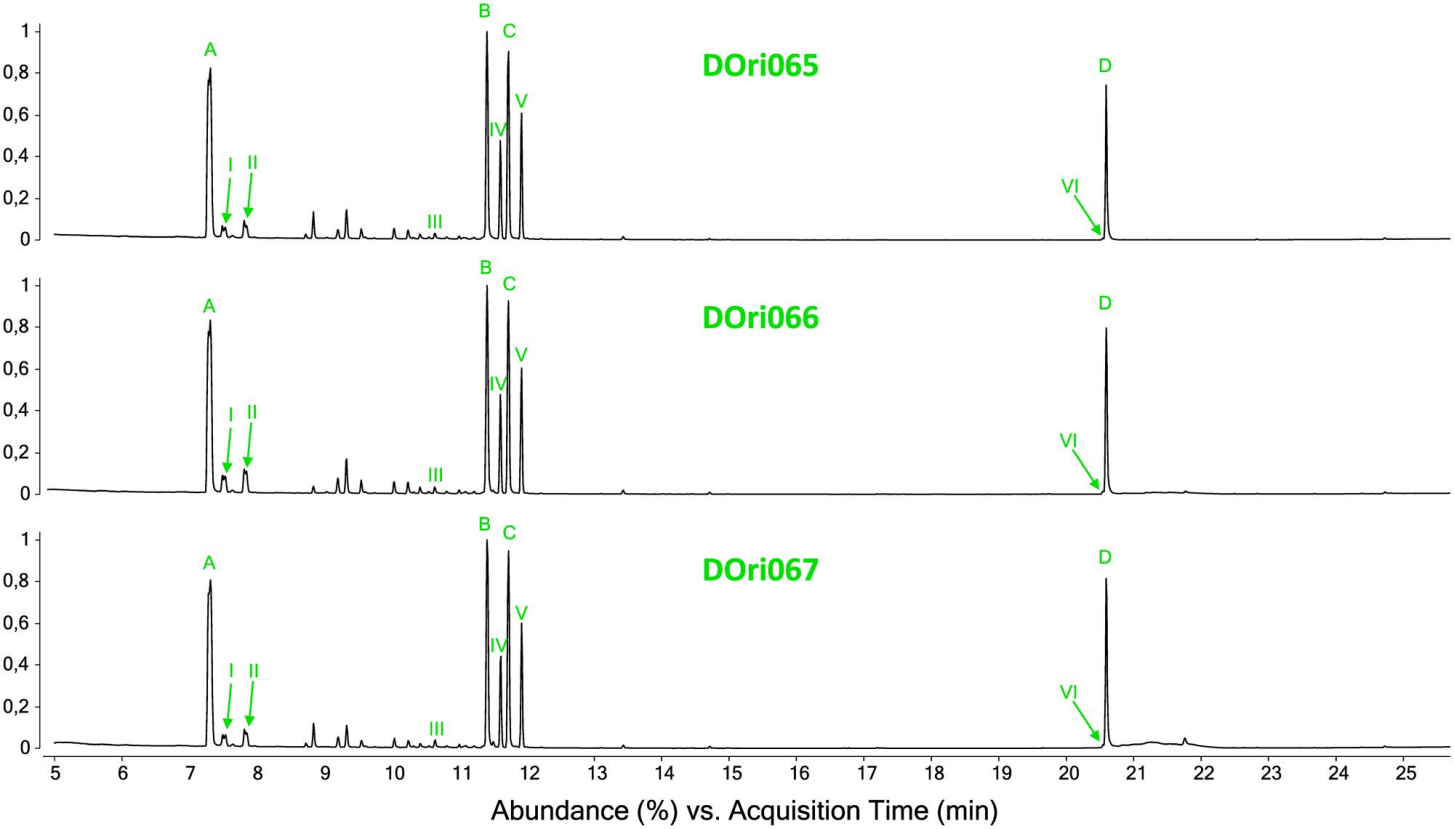
Chromatograms of the three samples of combined extraction (group 2). Letters (A–D) and numbers (I–IV) are referring to the compounds described in Raspotnig et al. [22]. These are: A) 2,3-dehydro-1,8-cineole, B) (4*R,*5*R*)- or (4*S,*5*S*)-*p*-1,8-methadien-5-yl formate, C) (4*R,*5*S*)- or (4*S,*5*R*)-*p*-1,8-methadien-5-yl formate, and D) 1,12-heneicosadiene (1,12-C_21:2_), I) *p*-menthane monoterpene C_10_H_14_ (isomer1), II) *p*-menthane monoterpene C_10_H_14_ (isomer 2), III) oxygenated *p*-menthane monoterpenoid C_10_H_16_O (probably *p*-1,8-menthadien-5-ol), IV) oxygenated *p*-menthane monoterpenoid C_10_H_18_O_2_ (isomer 1), V) oxygenated *p*-menthane monoterpenoid C_10_H_18_O_2_ (isomer 2), and VI) heneicosatriene (C_21:3_). Abundances of these substances were used to evaluate the chemical extraction during the combined extraction protocol.

The oil gland profiles from the combined extraction group exhibited a consistent pattern of compounds (Fig. 4, Supplementary file 5), showing the same four major compounds (A–D) reported by Raspotnig et al. [22]. Furthermore, the minor compounds (I–VI) identified in the aforementioned publication were also detected, with their amounts sometimes being higher than reported previously, most likely due to the pooled extraction of three individuals per sample. Additional minor components, not reported by Raspotnig et al. [22], were detected in our samples, but these were omitted from the calculation of relative abundance, pending independent verification.

## Discussion

Our method of combining the extraction of exocrine secretion and RNA is suitable for smallest arthropods, as demonstrated for individuals of less than 1 mm in body length. We, however, pooled three individuals of *N. palustris* to ensure that each sample contained enough RNA (200-300 ng in total) for sequencing. The latest generation of ultra-low RNA input library prep kits should make sequencing of RNA extracts from single individuals feasible. However, we here chose to pool samples to reduce potential biases due to age and condition of single specimens, as all individuals originated from litter samples, having unknown life histories, and were not reared in the lab. While the RNAseq data obtained for both the RNA- and combined extraction groups are highly consistent with respect to quality, the initial chemical extraction appeared to have a quantitative effect on the expression levels. For the extraction of secretions, a time of 15 min was chosen, which is a common extraction time in many chemical-ecological studies, ranging 3–30 min [35–37]. Sidova et al. [38] showed that RNA degradation in tadpoles of *Xenopus laevis* (Daudin, 1802) occurred within 5 min post-mortem, and different degradation rates were reported for different genes using qPCR. We propose experiments with shortened chemical extraction time to minimize these effects. We further note that the ideal minimal time for chemical extraction may be species specific and needs to be identified on a case-by-case basis. Previous studies found expression values to be stable for tissues that were kept on ice for hours [39]. On this basis, i.e. to avoid early RNA-degradation, we decided to cool the samples during chemical extraction on crushed ice, as this is easily available in every laboratory. The effect of methylene chloride on gene expression has been investigated on human leukemia cells [40]. Three hours in two different concentrations of DCM (20% & 50% inhibitory concentration of cell proliferation) altered the expression of several genes, most of which were related to immune response and apoptosis. Compared to our setting with pure methylene chloride, the alive exposure time for the mites during chemical extraction is much shorter. Our results are indicating that the extraction on ice did not have any effect on the chemical profiles obtained. Slight differences in the relative amount of the major and minor compounds compared to the recent investigation [22] were found. These are probably caused by using different GC columns (HP-5MS vs ZB-5) and the higher concentration of compounds in pooled samples, respectively.

Interestingly, the total amount of RNA was slightly higher in the samples of combined extraction. This was supported by the data from the Qubit and also by the measurement of the Tapestation. The quality of RNA appeared to be very good in either of the groups, RNA-only or combined, whereas the relative number of degraded fragments was a little bit higher in the samples of combined extraction, together with a lower RIN value. Visual investigation of the Tapestation assays showed slightly more fragments in the front of the combined 18S/28S-fragments-peak [41, 42]. Overall, the RNA quality in all of the samples was very high if compared to quality references as given by Gallego Romero et al. [43] who propose a conservative cutoff of a RIN between 7.9 and 6.4. Previous studies on rove beetles [18, 19] which have performed combined extraction comparable to our design reported the RIN value as quality measurements and performed RNAseq. However, no further formal analyses of the possible effects of this extraction method on the transcriptome or differential expression analysis have been presented.

All assembled transcripts appeared highly complete based on our BUSCO assessment, and no consistent differences in completeness were detected between the two groups. Only one sample showed a lower completeness level (sample DOri068), which was of the RNA-only group. Due to the high quality and equal amount of RNA as well as comparably high BUSCO-completeness of the samples, we conclude that chemical extraction prior to RNA extraction has no adverse effects on the overall quality of transcriptomes and our new approach is indeed suitable for phylotranscriptomic approaches.

When processing the RNAseq data through the Seq2Fun pipeline, PCA indicated clustering of samples according to the treatment group. This is reminiscent of the results obtained by Scholes & Lewis [44], who compared the impacts of different RNA isolation methods. However, no grouping of the two different extraction methods could be observed in any of the principal components (PC 1–5) alone, when considering the full set of transcripts using salmon and DEseq2.

**Figure 5.**
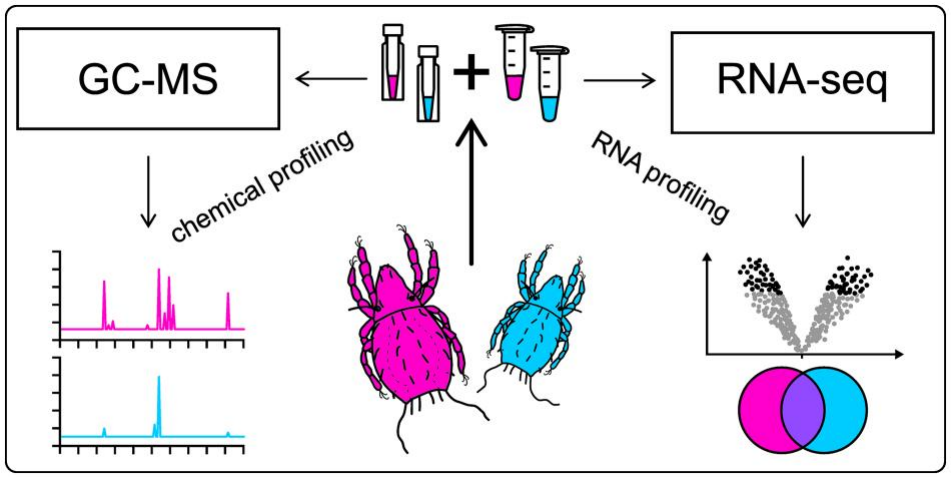
Study design for the proposed method. Both, RNA and chemical secretion, can be extracted from the same individuals and analyzed separately. This makes it possible to uncover correlations between the results of both methods and conduct specific study designs. Here, a hypothetic study using adults and juveniles of one species is presented. Potential other traits would be, e.g., gender, metabolic status, species.

Nevertheless, both analyses of expression levels, using the Seq2Fun-approach which mapped reads to a predefined protein database, and the salmon-pipeline which considered all transcripts assembled in the meta-transcriptome, respectively, identified a set of DEGs. Our combined extraction protocol thus appears to have some effect on the expression of certain genes, compared to direct RNA extraction without prior chemical extraction. Alternatively, it is possible that we have been indeed detected genuine biological variation between samples. While this cannot be fully ruled out, our random sampling and pooling strategy and the inclusion of three samples per group should have minimized the chances for these effects. Previous studies by Wang et al. [45] and Neymotin et al. [46] showed that transcripts encoding for functionally related genes have similar degradation rates. As such, we assert that as long as all samples within one study are processed in the same way, investigating expression profiles together with chemical profiles is feasible. Comparing expression profiles between samples processed by different procedures is generally not recommended unless the specific goal of the study is to investigate method specific biases as in Scholes & Lewis [44]. As certain functional groups of genes may be affected more or less strongly by degradation during sample processing, we advise caution when seeking to characterize full pathways or performing gene co-expression network analyses. Depending on the RNA extraction protocol, single individual lysates can be stored after homogenization until chemical profiles are investigated. Chemical profiling can be performed for individuals prior to pooling of material for actual RNA extraction, if RNA concentrations are otherwise insufficient for sequencing. Also, ready RNA extracts can be pooled according to the results generated from chemical profiling which allows targeting individuals of specific metabolic status, species, sex, or life stage. A hypothetic setup for such an investigation is found in Fig. 5. Also, a dissection of specific body parts or tissue from individuals after chemical extraction should be feasible and we even think of whole-animal multiplexed single-cell RNA-seq (WHAM-seq) to create a transcriptomic cell atlas [47].

In conclusion, our combined extraction method has the potential to (i) uncover candidate genes associated with exocrine compound synthesis, (ii) improve phylotranscriptomic and chemosystematic approaches where chemical data are mapped onto a phylogenetic tree, and (iii) allow targeted RNA-profiling for individuals of specific sex, life-stage, or metabolic status determined by initial individual chemical profiling.

## Supplementary Material

bpae054_Supplementary_Data

## Data Availability

Raw RNA-seq reads are deposited on sequence read archive under the accession numbers SRR29851544–SRR29851549 (Bioproject PRJNA1136254). Full assembly of all six samples together and further results are deposited with Zenodo (10.5281/zenodo.12756211).
