# Supplementary material for: Simple protocol for combined extraction of exocrine secretions and RNA in small arthropods
Source: Biol Methods Protoc. 2024 Jul 30;9(1):bpae054. doi: 10.1093/biomethods/bpae054 (PMC11316613; doi:10.1093/biomethods/bpae054)
Supplement: bpae054_Supplementary_Data [file bpae054_supplementary_data.zip › S05_GC-profiles.docx]

Table S.X. Chemical profiles* of *Nothrus palustris* used in the combined extraction method

| **Peak no.**^1^ | **RI^2^** | **DOri65E** | **DOri066E** | **DOri067E** |
| --- | --- | --- | --- | --- |
| **A** | 995 | 31,6 | 30,5 | 30,3 |
| **I** | 1008 | 1,1 | 1,6 | 1,1 |
| **II** | 1027 | 2,3 | 3,5 | 2,6 |
| **III** | 1213 | 0,5 | 0,6 | 0,7 |
| **B** | 1268 | 19,3 | 18,8 | 19,1 |
| **IV** | 1282 | 7,1 | 6,8 | 6,7 |
| **C** | 1291 | 16,9 | 16,5 | 16,8 |
| **V** | 1304 | 9,3 | 8,8 | 9,1 |
| **D** | 2082 | 11,9 | 13,0 | 13,7 |
| **VI** | 2079 | <1 | <1 | <1 |

* ^1^ Peak numbering follows the publication by Raspotnig et al. 2023, ^2^ Retention indices (RI) were calculated according to Van den Dool and Kratz 1963,
