# Supplementary figures and images for: Simple protocol for combined extraction of exocrine secretions and RNA in small arthropods
Source: Biol Methods Protoc. 2024 Jul 30;9(1):bpae054. doi: 10.1093/biomethods/bpae054 (PMC11316613; doi:10.1093/biomethods/bpae054)

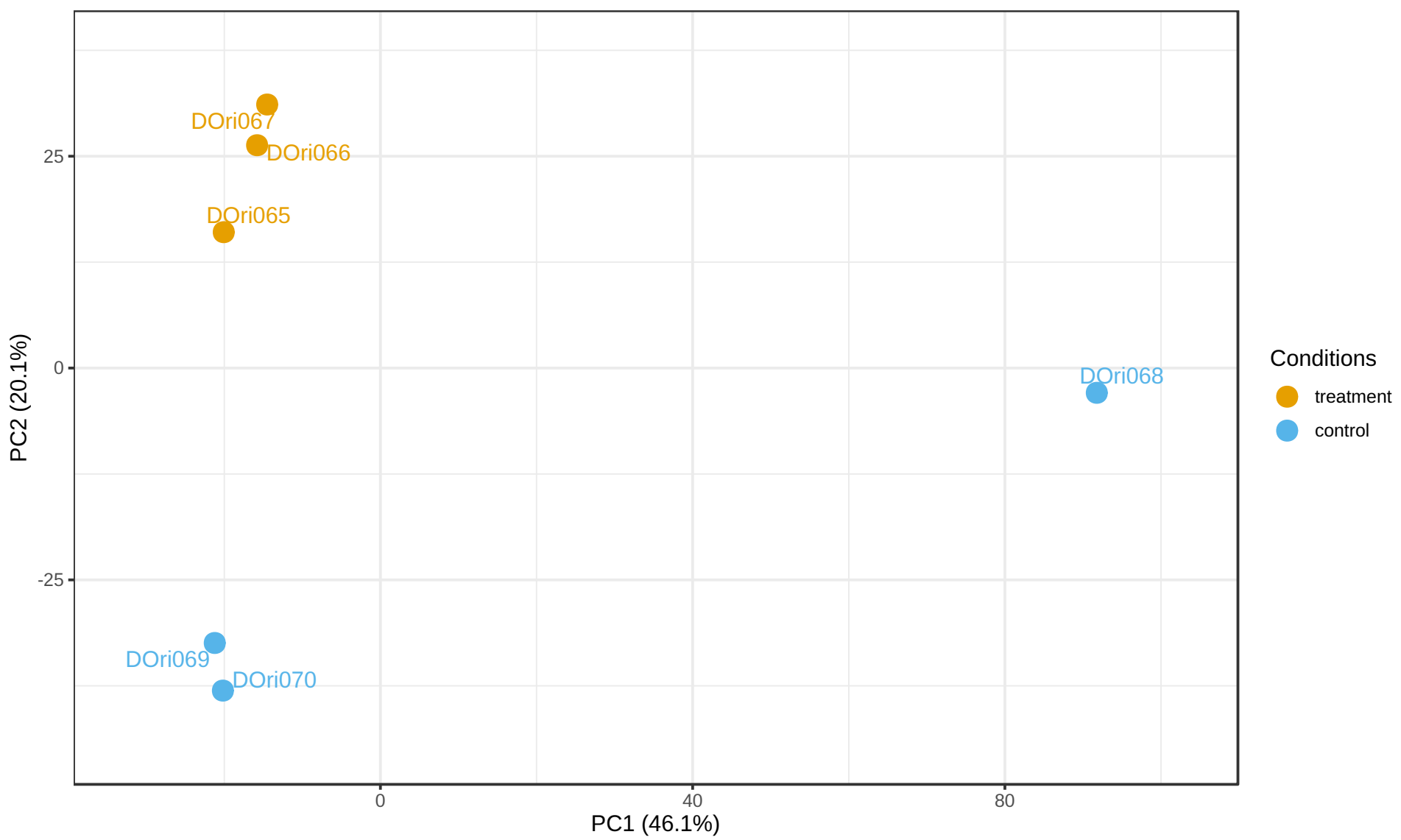

Supplement: bpae054_Supplementary_Data [file bpae054_supplementary_data.zip › S02_PC12seq2fun.pdf]
